# A cuproptosis-related genes signature associated with prognosis and immune cell infiltration in osteosarcoma

**DOI:** 10.3389/fonc.2022.1015094

**Published:** 2022-10-06

**Authors:** Weiguang Yang, Haiyang Wu, Linjian Tong, Yulin Wang, Qiang Guo, Lixia Xu, Hua Yan, Chengliang Yin, Zhiming Sun

**Affiliations:** ^1^ Clinical College of Neurology, Neurosurgery and Neurorehabilitation, Tianjin Medical University, Tianjin, China; ^2^ Department of Graduate School, Tianjin Medical University, Tianjin, China; ^3^ Department of Orthopaedics, Baodi Clinical College of Tianjin Medical University, Tianjin, China; ^4^ Tianjin Key Laboratory of Cerebral Vascular and Neurodegenerative Diseases, Tianjin Neurosurgical Institute, Tianjin Huanhu Hospital, Tianjin, China; ^5^ Faculty of Medicine, Macau University of Science and Technology, Taipa, Macao SAR, China; ^6^ Department of Orthopaedics, Tianjin Huanhu Hospital, Tianjin, China

**Keywords:** osteosarcoma, prognosis, cuproptosis, tumor microenvironment, bioinformatics analysis

## Abstract

Osteosarcoma (OS) is one of the most prevalent primary bone tumors at all ages of human development. The objective of our study was to develop a model of Cuproptosis-Related Genes (CRGs) for predicting prognosis in OS patients. All datasets of OS patients were obtained from the Therapeutically Applicable Research to Generate Effective Treatments (TARGET) database and Gene Expression Omnibus (GEO) database. We obtained the gene set (81 CRGs) related to cuproptosis by accessing the database and previous literature. All the CRGs were analyzed by univariate COX regression, least absolute shrinkage and selection operator (LASSO) COX regression analysis to screen for CRGs associated with prognosis in OS patients. Then these CRGs were used to construct a prognostic signature, which was further verified by independent cohort (GSE21257) and clinical correlation analysis. Afterward, to identify underlying mechanisms, Gene Ontology (GO) analysis and Kyoto Encyclopedia of Genes and Genomes (KEGG) analysis were used for the high-risk group by using the GSEA method. The association between the prognostic signature and 28 types of immune infiltrating cells in the tumor microenvironment was assessed. Ultimately, Lipoic Acid Synthetase (LIAS) (HR=0.632, P=0.004), Lipoyltransferase 1 (LIPT1) (HR=0.524, P=0.011), BCL2 Like 1 (BCL2L1/BCL-XL) (HR=0.593, P=0.022), and Pyruvate Dehydrogenase Kinase 1 (PDK1) (HR=0.662, P=0.025) were identified. Subsequently, they were used to calculate the risk score and build a prognostic model. In the training cohort, risk score (HR=1.878, P=0.003) could be considered as an independent prognostic factor, and OS patients with high-risk scores showed lower survival rates. Biological pathways related to substance metabolism and transport were enriched. There were significant differences in immune infiltrating cells in the tumor microenvironment. All in all, The CRGs signature is related to the tumor immune microenvironment and could be used as a credible predictor of the prognostic status in OS patients.

## Introduction

OS is the main form of malignant bone cancer, predominantly affecting adolescents in a period of rapid growth. It occurs mostly in the long epiphysis and has a poor prognosis. The disability rate is high if OS is not treated properly. OS patients are prone to recurrence and metastasis, especially lung metastasis, which is the primary reason for their bad prognosis. Patients with high-risk OS have less than a 30% chance of surviving more than five years (1, 2). Recently, due to the introduction of adjuvant and neoadjuvant chemotherapy regimens, the prognosis and survival rate of OS patients have been significantly improved. However, the total survival in patients with lung metastases or recurrence remains bad and has not improved significantly (3, 4). The data of previous studies showed that OS patients with lung metastases had a 30% probability of surviving longer than five years, while OS patients without lung metastases had a 70% probability of surviving longer than five years (5, 6). Thus, understanding the pathogenesis and identifying a new signature that predicts the prognosis of OS patients is crucial for improving outcomes. Copper is an extremely important metal element involved in all kinds of biological procedures in the human body. Recent studies have shown that compared with healthy people, excess copper ions were found in the blood and tumor tissues of patients with cancer, for example, colorectal, breast, lung, prostate and brain cancer (7–9). In addition, copper can cause various ways of cell death through different mechanisms, for example, apoptosis and autophagy, accumulation of reactive oxygen species, proteasome inhibition, and anti-angiogenesis (10). Therefore, imbalances in copper homeostasis may affect cancer development and progression and cause irreversible damage (11). Mutations that lead to copper overload can have serious consequences. However, by rationally controlling intracellular copper levels, tumor cells can be selectively killed (8). Recently, a novel form of cell death was discovered, elevated copper levels in mitochondria induce this form of cell death. Excess intracellular copper leads to abnormal accumulation of fatty acylated proteins and a decrease of Fe/S proteins by combining fatty acylated elements of the tricarboxylic acid cycle, triggering proteotoxic stress responses that ultimately lead to cell death (12). In our study, we established a model of Cuproptosis-Related Genes (CRGs) for predicting prognosis in OS patients and to observe its association with immune infiltrating cells in the tumor microenvironment. Identification of CRGs may provide a new method to predict outcomes in OS patients.

## Materials and methods

### Data collection

Clinical data and RNA sequencing data on patients with OS were obtained from the TARGET (https://www.ocg.cancer.gov/programs) and GEO databases (https://www.ncbi.nlm.nih.gov/geo/). The datasets or patient samples selected in this study should meet the following conditions: (1) OS was diagnosed histologically in all samples; (2) the dataset contains both metastatic and no-metastatic patients; (3) the dataset has necessary prognostic-related information. There were 4 patients in the TARGET dataset and 1 patient in the GSE21257 dataset with incomplete clinical characteristics or follow-up information, we rule they out. Finally, 84 OS patients in Target were selected as the training cohort, and 52 OS patients in GSE21257 were selected as the validation cohort. Gene set associated with cuproptosis were obtained by accessing databases and previous literature (Additional Documents 1).

### Screening of prognosis-associated genes in OS patients, construction and validation of the prognostic model

First, a univariate COX regression analysis was performed to determine the prognosis-associated CRGs in patients with OS (P<0.05), whose dataset was obtained from the TARGET database. Then use the “glmnet” package to conduct LASSO COX analysis on these CRGs, and the minimum lambda is defined as the optimal value. Finally, four CRGs were determined to build the prognostic model. The obtained risk scores and other clinical characteristics were again subjected to univariate COX and multivariate COX regression analysis, and the “timeROC” and “survminer” packages were used to draw ROC curves (13) and Kaplan-Meier survival curves, respectively, to discern differences between high-risk group and low-risk group. Subsequently, the nomogram and its calibration curve (14), and the Kaplan-Meier survival curve between each subgroup were drawn to verify our model accuracy and independence.

### Functional enrichment analysis and immune cell infiltration analysis

GO and KEGG enrichment analysis was used for samples from the high-risk group of the training cohort and validation cohort using the GSEA method (15). Feature enrichment analysis was done by the “clusterProfiler” and “enrichplot” packages (16). A dataset including 28 immune cells and related genes was obtained from the literature (17), and the enrichment of 28 immune infiltrating cells was evaluated by single-sample gene set enrichment analysis (ssGSEA). Overlapping items with the same trend were considered immune signature changes.

### Statistical analysis and bioinformatics analysis

All the above statistical and bioinformatic analyses were implemented by R version 4.1.2 (2021-11-01) and corresponding R software packages. Differences between two or more groups were tested by Student’s t-test and one-way analysis of variance (ANOVA), respectively. Differences were considered statistically significant if P<0.05.

## Results

### Construction of a Cuproptosis-related prognostic model

**Figure 1:**
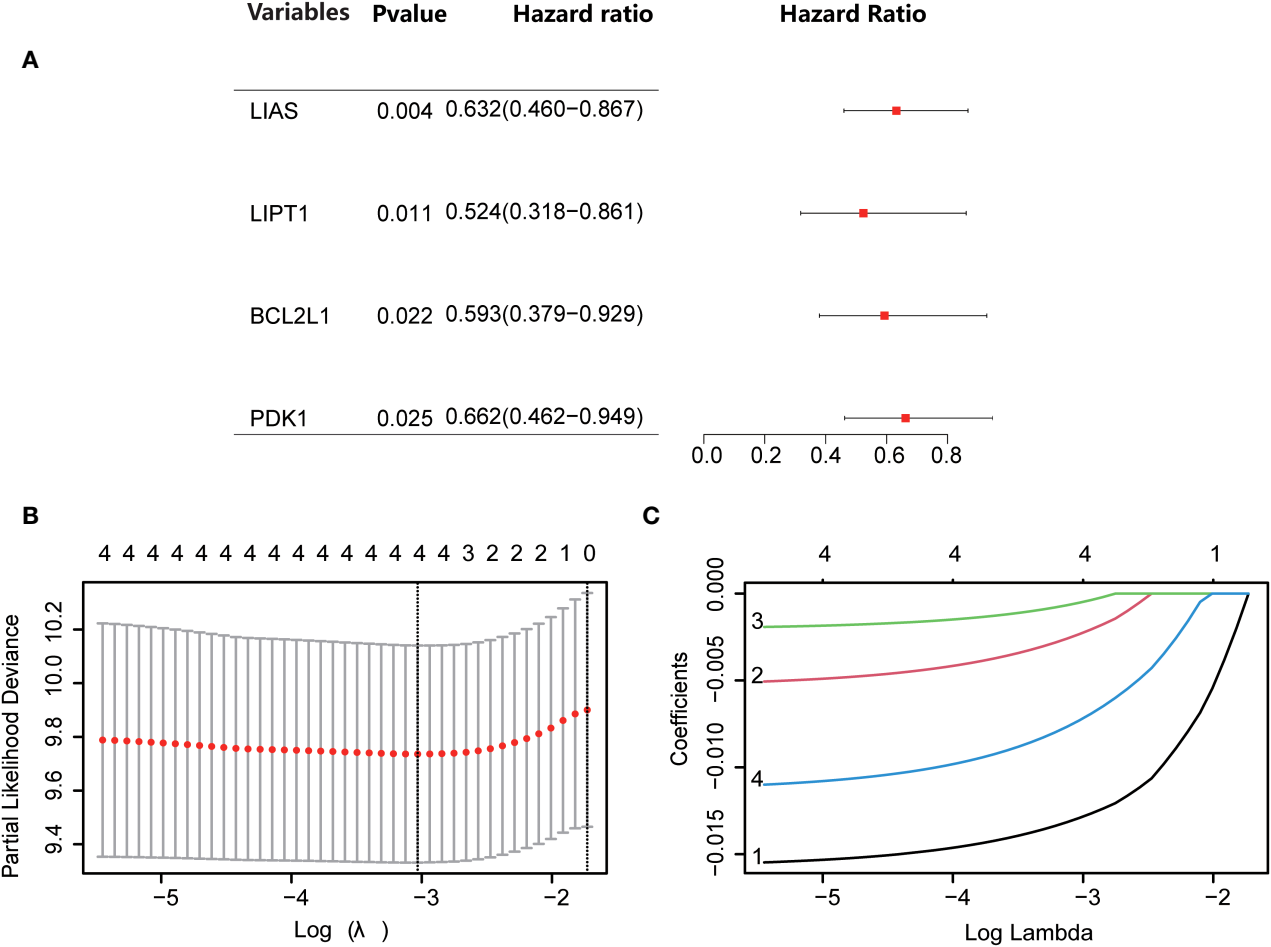
Screening for Cuproptosis-Related Genes. **(A)** Forest plot showing the results of univariate Cox regression analyses. **(B, C)** LASSO analysis with minimal lambda.

**Figure 2:**
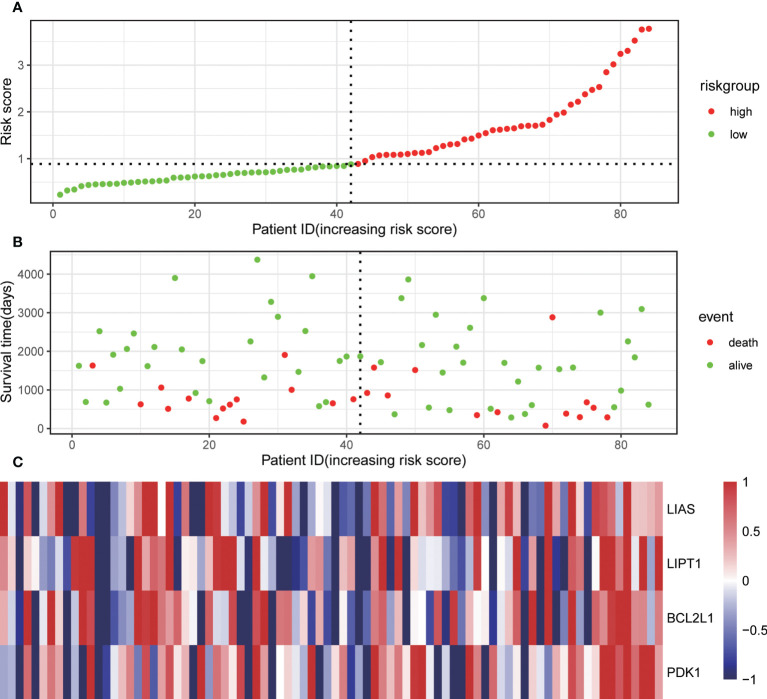
Associations of the risk score with the expression levels of four Cuproptosis-Related Genes included in the risk model. **(A)** Dot plot of risk score. **(B)** Dot plot of survival. **(C)** Heat map of the expression levels of the four genes.

**Figure 3:**
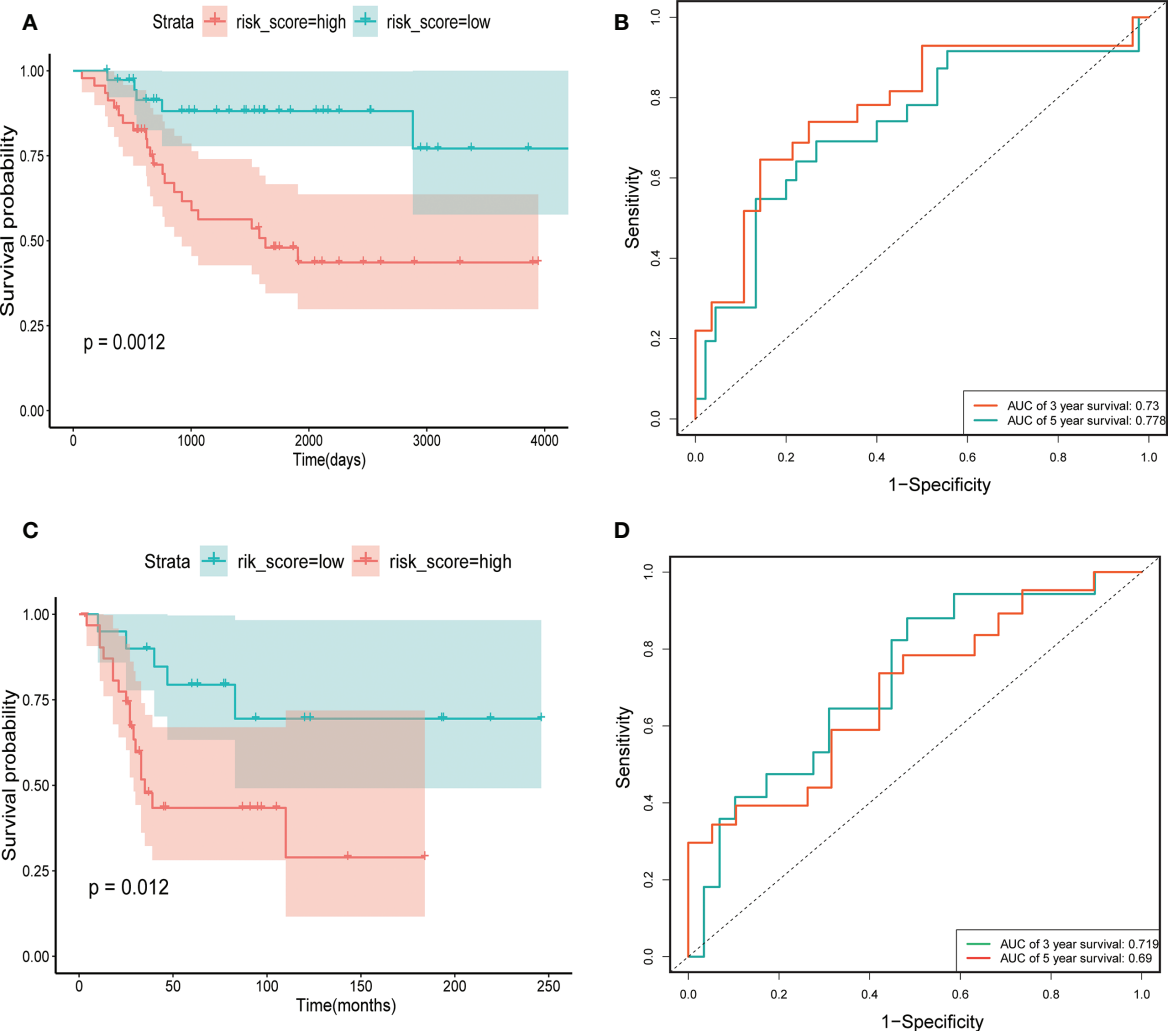
The construction and verification of risk signature. **(A)** Training cohort survival analysis. **(B)** Training cohort ROC curve analysis. **(C)** Validation cohort survival analysis. **(D)** Validation cohort ROC curve analysis.

Through univariate Cox regression, 4 relevant CRGs were screened and preprocessed from 84 OS samples downloaded and preprocessed from TARGET database, among which LIAS (P=0.004, HR=0.632), LIPT1 (P=0.011, HR =0.524), BCL2L1 (P=0.022, HR=0.593), PDK1 (P=0.025, HR=0.662) were protective factors (**Figure 1A**). The LASSO COX regression method was used for further analysis, and the related CRGs obtained were still these four genes (**Figures 1B, C**). Then, we use the “predict” function to calculate the risk score and use the “surv_cutpoint” function of the “survminer” package to select the optimal cutoff value to divide the OS samples into high-risk and low-risk groups. The risk score plot (**Figure 2A**), survival status plot (**Figure 2B**), and heatmap (**Figure 2C**) showed a good distinction between the low-risk and high-risk groups. KM survival analysis showed that the high-risk group was an apparently poor prognosis (P=0.0012) (**Figure 3A**). Additionally, the training cohort ROC curve showed that the results were credible, the 3-year Area Under Curve (AUC) is 0.73 and 5-year AUC is 0.778 (**Figure 3B**). Analogous results were obtained in the KM survival analysis of the validation cohort (P=0.012) (**Figure 3C**), and 3-year AUC is 0.719 and 5-year AUC is 0.69 (**Figure 3D**).

### Validation of the independence of the prognostic model

**Figure 4:**
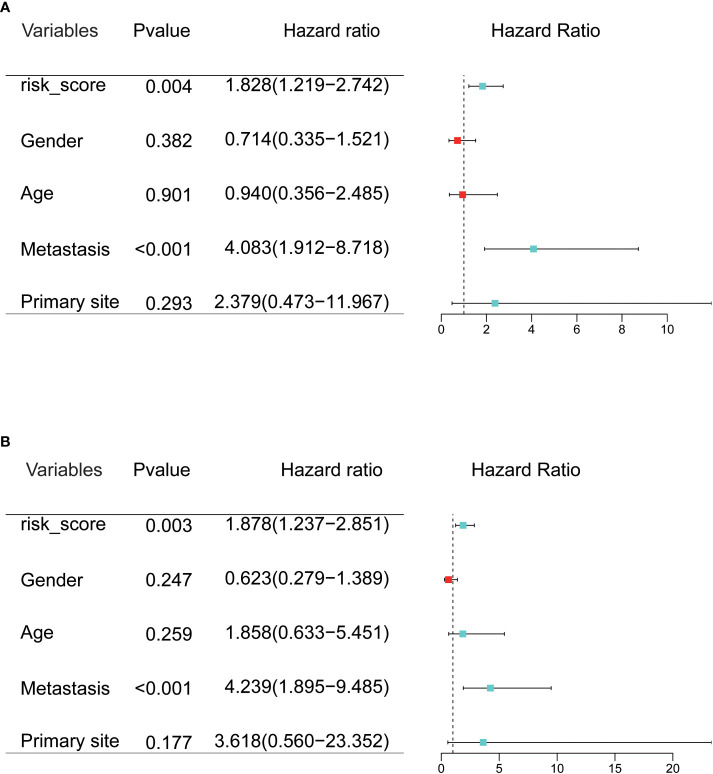
**(A, B)** Univariate and multivariate Cox regression analysis was used to evaluate the contribution of each variable to the OS patients.

**Figure 5:**
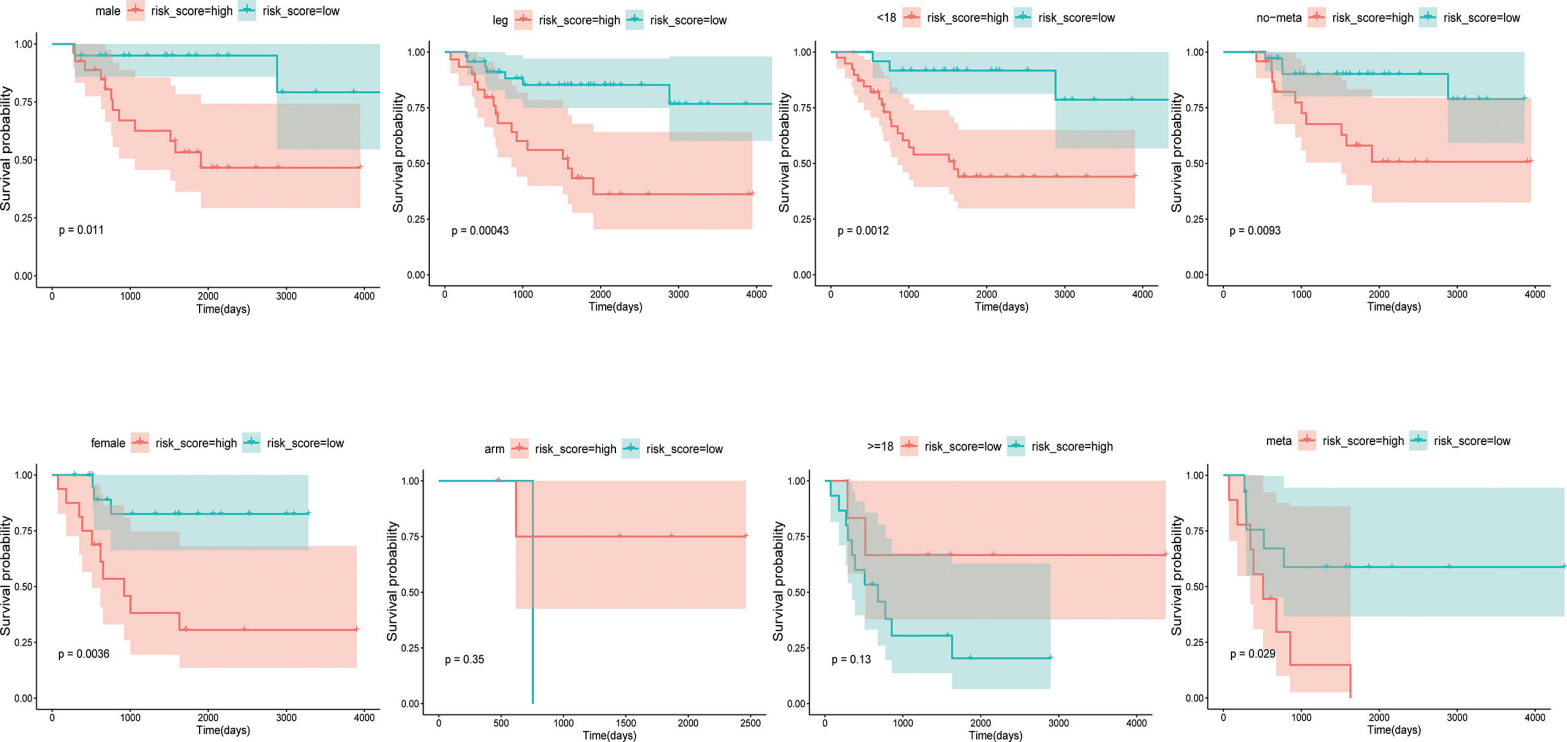
Kaplan-Meier curves of OS patients in different clinical subgroups.

To evaluate the prognostic value of CRGs signatures in clinical applications, univariate and multivariate COX regression analyses were performed again to assess whether the risk score or other indicators such as sex, age, metastatic status, and primary site could assume independent predictive indicators for OS patients. Univariate COX regression analysis results showed that risk score (P=0.004, HR=1.828) and metastasis (P<0.001, HR=4.083) correlated with the overall survival of OS patients (**Figure 4A**). And multivariate COX regression analysis results showed that the risk score (P=0.003, HR=1.878) and metastasis (P<0.001, HR=4.239) could serve as independent prognostic indicators to predict the overall survival of OS patients (**Figure 4B**). Moreover, we researched the relation between risk score and clinical characteristics and assessed the independence of the established prognosis model by subgroup KM survival analysis (**Figure 5**). There were no associations between risk scores and these clinical characteristics of age, sex, primary site, as well as metastatic status. High-risk patients of each subgroup had a poor prognosis. These results suggested that the constructed risk model had apparent independence in foresting the prognosis of OS.

### Nomogram construction and validation

**Figure 6:**
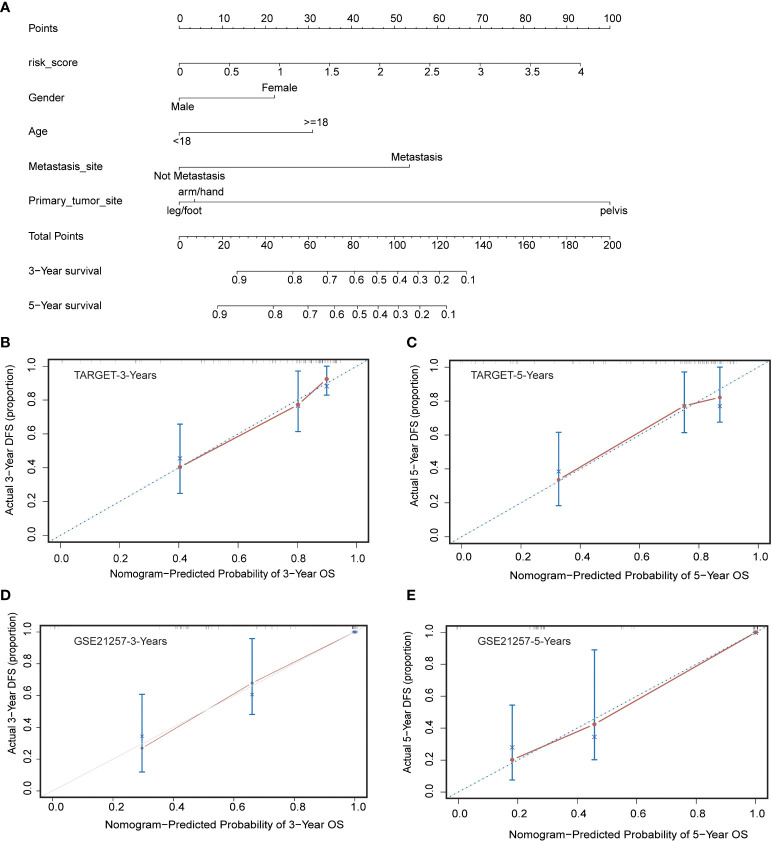
Construction and calibration of nomogram. **(A)** Nomogram integrating risk score and clinical characteristics **(B, C)** Calibration of the nomogram at 3-year and 5-year survival in the training cohort. **(D, E)** Calibration of the nomogram at 3-year and 5-year survival in the validation cohort.

To better predict 3- and 5-year survival in OS patients, we drew a nomogram that integrated risk score and clinical characteristics including risk score, sex, age, metastatic status, and primary site (**Figure 6A**). As shown in the figure, by assigning a score to each item according to the actual situation, the patient can obtain a total score that predicted survival at 3 and 5 years. The predictive accuracy of the nomogram was then verified in the training cohort and the validation cohort (**Figures 6B–E**). The solid red line indicated the actual survival status and the dashed line indicated the optimized survival status, which showed an excellent fit between the actual value and optimized value in the training and validation cohort.

### Gene function enrichment analysis

**Figure 7:**
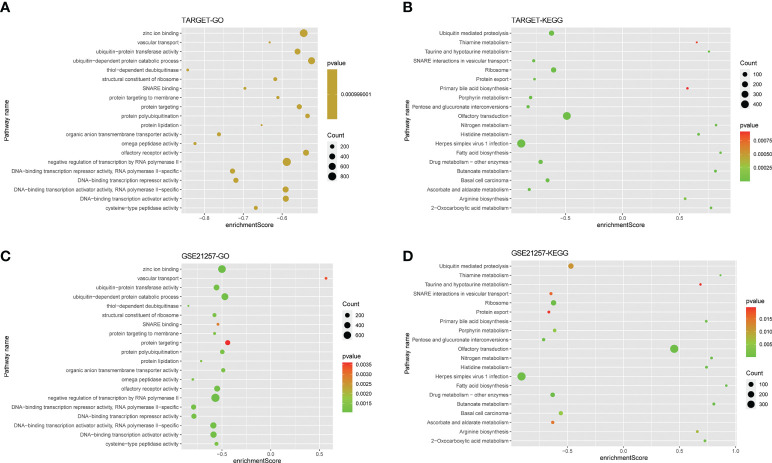
Gene function enrichment analysis. **(A, B)** The bubble plot showing the top twenty KEGG analysis enriched signaling pathways and GO analysis enriched biological processes in the training cohort. **(C, D)** The bubble plot showing the top twenty KEGG analysis enriched signaling pathways and GO analysis enriched biological processes in the validation cohort.

GO and KEGG of the GSEA method enrichment analysis based on the genes of patients in the TARGET high-risk group, a total of 148 biological processes (Additional Documents 2) and 25 biological pathways (Additional Documents 3) were enriched (P<0.05, FDR<0.05), most of which were related to substance metabolism and transport. In the biological pathways of the KEGG analysis, including pentose and glucuronic acid interconversion, fatty acid biosynthesis, protein export, etc. In the biological processes of the GO analysis, including lipoprotein metabolism process, protein acylation, protein lipidation, etc. As shown in **Figures 7A–D**, we selected the top 20 to draw bubble charts.

### Analysis of immune cell infiltration

**Figure 8:**
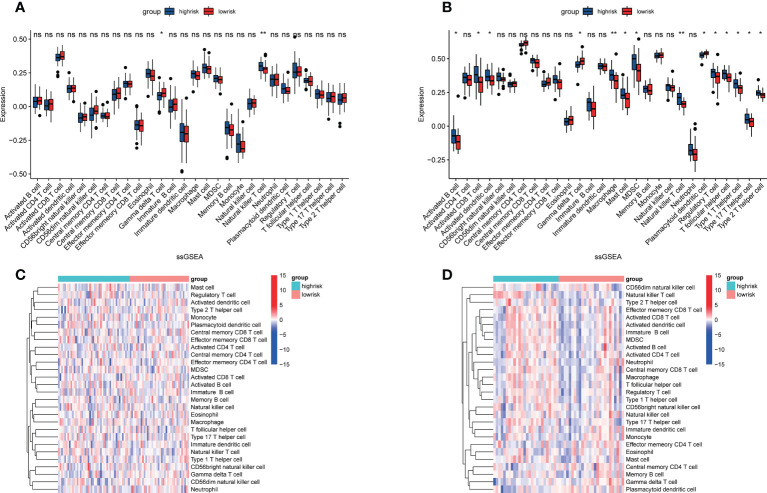
Assessment of immune cell infiltration in OS tissue from high- and low-risk patients using the ssGSEA method. **(A, B)** Differences in immune cell infiltration between high- and low-risk OS patients. *p<0.05; **p<0.01; NS, no significance. **(C, D)** Heatmap depicting the enriching level of 28 immune related cells evaluated by ssGSEA algorithm.

**Figure 9:**
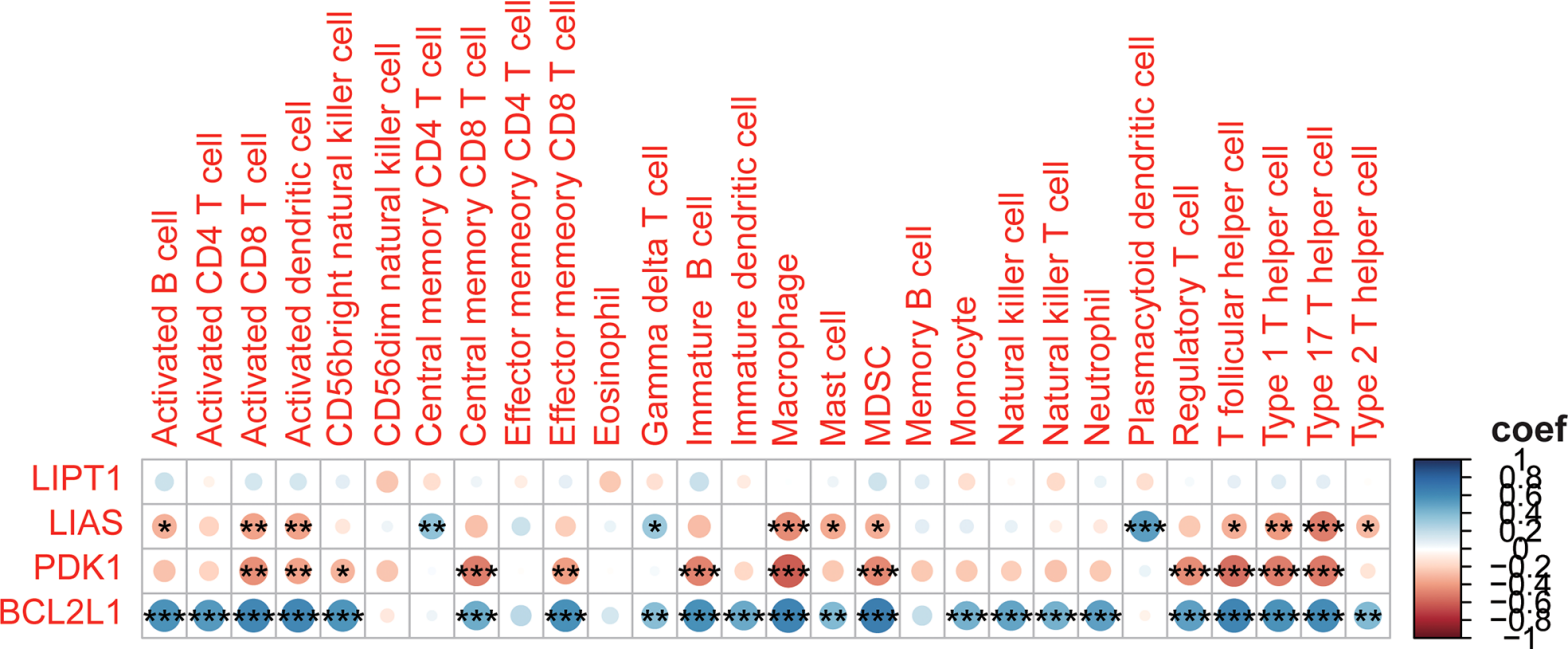
Correlation between the 4 CRGs and immune cell infiltration. *p<0.05; **p<0.01; ***p<0.001.

To further understand the relationship between CRGs and immune infiltrating cells, we performed ssGSEA analysis on datasets from TARGET and GEO (**Figure 8**). The results of ssGSEA analysis showed that, in the TARGET dataset, Gamma delta T cells were down-regulated and Natural killer T cells were up-regulated in patients with high-risk scores. In the GEO dataset, apart from the above two changes, activated B cell, activated CD8 T cell, activated dendritic cell, macrophage, mast cell, MDSC, regulatory T cell, T follicular helper cell, type 1 T helper cell, type 17 T helper cell, and type 2 T helper cell were also up-regulated in OS patients with high-risk scores, and the plasmacytoid dendritic cell was down-regulated. Correlation analyses between CRGs and infiltrating immune cells suggested that the expression levels of LIAS and PDK1 were negatively associated with the levels of most immune infiltrating cells. The expression level of BCL2L1 was positively associated with the levels of most immune infiltrating cells (**Figure 9**).

## Discussion

OS is almost the most aggressive bone cancer in children and adolescents worldwide (18). Although there were various treatment strategies such as surgery, radiotherapy, chemotherapy and neoadjuvant chemotherapy, the 5 years survival rate of OS patients had only a small improvement over the past 35 years. Thus, the development of individualized diagnostic and therapeutic strategies is imminent (19, 20). In most life forms, metals are important parts of most enzymatic functions and are closely related to most basic biological processes. There are some main functions of metals: to provide structural support, act as cofactors for enzymes, and mediate electron transfer. Moreover, studies have suggested that metal elements were closely linked to the progress of cancers (21, 22). Cuproptpsis, an unconventional cell death mechanism associated with fatty acylation of proteins in the TCA cycle, may suggest novel approaches to exploit copper toxicity for tumor therapy. Copper ionophores and copper chelators have already been used in anticancer therapy, such as disulfiram, dithiocarbamates, elesclomol, trientine, tetrathiomolybdate (23–26).

In this study, four CRGs (LIAS, LIPT1, PDK1, BCL2L1) associated with the prognosis of OS were identified through the GEO and TARGET databases. Then a prognostic model was established and a risk score was constructed for prediction and verification. Of the four genes identified, lipoic acid (LA) is a key cofactor in the mitochondrial 2-keto acid dehydrogenase and glycine cleavage system, which is involved in the TCA cycle and mitochondrial energy metabolism and amino acid catabolism. Fatty acyl transfer encoded by LIPT1 Enzyme 1 transfers lipid salts to the E2 subunit of 2-keto acid dehydrogenase and is involved in lipoic acid regulation LIPT1 deficiency which was shown to inhibit TCA cycle metabolism (27–29). LIAS encoded a component of the lipoic acid pathway and synthesized a potent antioxidant called alpha-LA in the mitochondria (30). While the deficiency of LIAS blocks lipid acylation of the protein would lead to the insensitivity of cells to copper-induced cell death (31). Mechanistically, PDK1 was the hypoxia-inducible factor-1α (HIF-1α) targeted antagonist of pyruvate dehydrogenase (PDH), an important speed-limited enzyme in the TCA cycle. In the absence of oxygen, the conversion of pyruvate to acetyl-CoA is inhibited because of PDK1-dependent inhibition of PDH, thereby decreasing the number of glucose-derived pyruvates participating in the TCA cycle (32, 33). Furthermore, copper bounds PDK1, which then activated the downstream substrate AKT and led tumor development (34). There are four N-donor polypyridyl copper (II) complexes: [Cu(mono-CN-PIP)2]^2+^, [Cu(tri-OMe-PIP)2]^2+^, [Cu(di-CF3-PIP)2]^2+^ and [Cu(DPPZ)2]^2+^), they could cause an abnormal increase in cellular reactive oxygen species (ROS) excess by downregulating BCL2L1, leading to cell death. This may be the plausible mechanism behind its anticancer properties (35). In addition, previous studies revealed that high expression of LIAS and LIPT1 were favorable for the prognosis of clear cell renal cell carcinoma (36), and LIPT1 could also be considered as an indicator of favorable prognosis in melanoma (37). LIPT1 was related to a better prognosis of urothelial cancer (38). Previous studies have found that PDK1 is overexpressed in multiple myeloma (MM) (39), acute myeloid leukemia (AML) (40), breast invasive carcinoma (BRCA) (41), and OS (42).

The independence of the prognosis model was well validated in the training cohort by univariate and multivariate cox regression analysis, and subgroup KM survival analysis. In addition, metastatic status was also an independent factor influencing the outcome of OS patients. To further forecast the prognosis of these patients, we drew a nomogram containing risk score, gender, age, metastatic status, and primary site. The prognostic prediction performance of the nomogram was well demonstrated with 3-year and 5-year survival rates in the training cohort, further demonstrating the predictive function of the prognostic model.

It is commonly held that immune cell infiltration in the TME modulates all kinds of tumor characteristics, such as malignancy of the tumor and metastatic ability (43–45). We used the ssGSEA method to analyze the structure of immune infiltrating cells in OS’s TME. We found that higher levels of natural killer T cells, activated B cells, activated CD8 T cells, and activated dendritic cells, macrophage, mast cell, MDSC, regulatory T cell, T follicular helper cell, type 1 T helper cell, type 17 T Helper cells, type 2 T helper cells, and low levels of Gamma delta T cells plasmacytoid dendritic cells are unfavorable for the prognosis of patients with OS. These results suggested that CRGs may influence the aforementioned immune cell infiltration and have an important effect on the metastasis and progression of OS. It has been shown that Natural killer T cells and type 2 T helper cells are associated with poor prognosis in patients with OS (46). Clinical research has shown that Natural killer T cells in the blood of OS patients are significantly lower than that of normal people (47). Based on the correlation analysis, we concluded that LIAS, PDK1, and BCL2L1 are closely related to infiltrating immune cells. The antiapoptotic oncoprotein BCL2 plays a key role in the development and outcome of the tumor, lymphocyte growth, and immune system adjustment (48). PDK1 is significant for the most of immune cell development and function, containing T cells, B cells, and NK cells (49–54). The expression of LIPT1 was positively correlated with CD8 T cells and macrophages in Uterine Corpus Endometrial Carcinoma (55). The expression of LIPT1 is associated with the infiltration of many immune cells in various cancers (56). Nevertheless, the detailed mechanism of the influences of CRGs on immune cell infiltration is unclear and deserves further exploration.

Hitherto, we are the first to investigate the relationship between CRGs and the prognosis of OS. Cell death is extremely important in cancer research and underlies the origin and development of tumors (57). Cuproptosis is a newly found way of cell death that relies on mitochondrial respiration and is different from other forms of cell death such as pyroptosis, ferroptosis and autophagy (21, 22). This special mechanism may lead to a new idea for the treatment of OS. Our study has some shortcomings. Firstly, we could not further explore the functions of CRGs in OS progression due to the lack of information such as tumor stage. Secondly, our results were not further validated by experiments and were derived from bioinformatics analysis. Thirdly, the data used in our research are not our own but are obtained from public databases. If the prognostic value of CRGs in OS is to be further proved, more prospective studies are demanded.

## Conclusion

All in all, we established and validated a newly CRGs prognostic signature. And our results proved it was associated with OS prognosis. Additionally, our study also found that CRGs could influence the immune cells in the TME and further affect the OS development, which provides new clues for the exploration of immunotherapeutic approaches for OS patients. However, more relevant researches should be conducted to further quest the connection between cuproptosis and osteosarcoma, which is expected to lead to improved treatment of OS patients.

## Data availability statement

The datasets presented in this study can be found in online repositories. The names of the repository/repositories and accession number(s) can be found in the article/**Supplementary Material**.

## Author contributions

WY, HW, LT, HY, CY, and ZS designed the study. WY, HW, LT, YW, QG, and LX collected the data. WY, HW, LX, HY, and ZS analyzed the data and drafted the manuscript. CY, ZS, and HY revised and approved the final version of the manuscript. All authors read and approved the submitted version.

## Funding

This work was supported by the Tianjin Municipal Health Bureau (grant numbers 14KG115) and the Key Program of the Natural Science Foundation of Tianjin (grant numbers 20JCZDJC00730).

## Acknowledgments

The authors thank Dr. Changshan Wan and “home-for-researchers (www.home-for-researchers.com)” for their help in polishing our English writing.

## Conflict of interest

The authors declare that the research was conducted in the absence of any commercial or financial relationships that could be construed as a potential conflict of interest.

## Publisher’s note

All claims expressed in this article are solely those of the authors and do not necessarily represent those of their affiliated organizations, or those of the publisher, the editors and the reviewers. Any product that may be evaluated in this article, or claim that may be made by its manufacturer, is not guaranteed or endorsed by the publisher.
